# Effectiveness of Shared Decision-making Training Programs for Health Care Professionals Using Reflexivity Strategies: Secondary Analysis of a Systematic Review

**DOI:** 10.2196/42033

**Published:** 2022-12-07

**Authors:** Ndeye Thiab Diouf, Angèle Musabyimana, Virginie Blanchette, Johanie Lépine, Sabrina Guay-Bélanger, Marie-Claude Tremblay, Maman Joyce Dogba, France Légaré

**Affiliations:** 1 Canada Research Chair in Shared Decision Making and Knowledge Translation (Tier 1) Quebec, QC Canada; 2 VITAM - Centre de recherche en santé durable Centre intégré universitaire de santé et services sociaux de la Capitale-Nationale Quebec, QC Canada; 3 Department of Community Health Faculty of Nursing and Faculty of Medicine Université Laval Quebec, QC Canada; 4 Department of Human Kinetic and Podiatric Medicine Université du Québec à Trois-Rivières Trois-Rivières, QC Canada; 5 Office of Education and Continuing Professional Education Université Laval Quebec, QC Canada; 6 Department of Family Medicine and Emergency Medicine Faculty of Medicine Université Laval Quebec, QC Canada

**Keywords:** shared decision-making, reflexivity, training, health care professionals, implementation

## Abstract

**Background:**

Shared decision-making (SDM) leads to better health care processes through collaboration between health care professionals and patients. Training is recognized as a promising intervention to foster SDM by health care professionals. However, the most effective training type is still unclear. Reflexivity is an exercise that leads health care professionals to question their own values to better consider patient values and support patients while least influencing their decisions. Training that uses reflexivity strategies could motivate them to engage in SDM and be more open to diversity.

**Objective:**

In this secondary analysis of a 2018 Cochrane review of interventions for improving SDM by health care professionals, we aimed to identify SDM training programs that included reflexivity strategies and were assessed as effective. In addition, we aimed to explore whether further factors can be associated with or enhance their effectiveness.

**Methods:**

From the Cochrane review, we first extracted training programs targeting health care professionals. Second, we developed a grid to help identify training programs that used reflexivity strategies. Third, those identified were further categorized according to the type of strategy used. At each step, we identified the proportion of programs that were classified as effective by the Cochrane review (2018) so that we could compare their effectiveness. In addition, we wanted to see whether effectiveness was similar between programs using peer-to-peer group learning and those with an interprofessional orientation. Finally, the Cochrane review selected programs that were evaluated using patient-reported or observer-reported outcome measurements. We examined which of these measurements was most often used in effective training programs.

**Results:**

Of the 31 training programs extracted, 24 (77%) were interactive, among which 10 (42%) were considered effective. Of these 31 programs, 7 (23%) were unidirectional, among which 1 (14%) was considered effective. Of the 24 interactive programs, 7 (29%) included reflexivity strategies. Of the 7 training programs with reflexivity strategies, 5 (71%) used a peer-to-peer group learning strategy, among which 3 (60%) were effective; the other 2 (29%) used a self-appraisal individual learning strategy, neither of which was effective. Of the 31 training programs extracted, 5 (16%) programs had an interprofessional orientation, among which 3 (60%) were effective; the remaining 26 (84%) of the 31 programs were without interprofessional orientation, among which 8 (31%) were effective. Finally, 12 (39%) of 31 programs used observer-based measurements, among which more than half (7/12, 58%) were effective.

**Conclusions:**

Our study is the first to evaluate the effectiveness of SDM training programs that include reflexivity strategies. Its conclusions open avenues for enriching future SDM training programs with reflexivity strategies. The grid developed to identify training programs that used reflexivity strategies, when further tested and validated, can guide future assessments of reflexivity components in SDM training.

## Introduction

### Background

There is increasing recognition of the ethical imperative to support patients to be engaged in their care, especially in health-related decisions. Shared decision-making (SDM) is a collaborative process whereby health care professionals support patients in making decisions that are informed by the best evidence and by what matters to them [1]. SDM improves the health care experiences of patients and health care professionals and leads to better health care processes, patient outcomes, and lower health costs [2-5]. SDM is the best practice for informed consent and is fundamental to patient- and family-centered care [6]. However, SDM has not yet been widely implemented in clinical practice because of several perceived barriers [7]. In some contexts, SDM implementation is encouraged by health policies, but certain challenges related to patients, patient–health care professional relationships, and organizational factors remain in the way of its concrete adoption [8].

### Reflexivity

Training programs for health care professionals [9,10] are believed to be crucial to the implementation of SDM. However, SDM training programs for health care professionals are highly heterogeneous [10], and we still do not know what makes them effective [9]. One promising approach is reflexivity, which has been shown to increase health care professionals’ willingness to be more engaged in the health care offer and collaborate with other professionals [11]. SDM training programs that use reflexivity strategies have the potential to be more effective than those that do not [12].

Reflexivity is a form of learning based on reflection on one’s experiences applied in a professional or interprofessional context [13]. Concepts related to reflexivity are found in various disciplinary fields under different names. Writing about *reflective practice* (a practice based on reflexivity), Schon [14] states that the concept entails critical thinking concerning the actions and stances one takes [14,15]. In health care practices, being reflexive can mean different things: acknowledging and questioning the power dynamics implicit in a health care encounter; identifying the assumptions that underlie a health care situation; or examining the influences, such as values and beliefs, that shape health care practices [13].

On the basis of this multiplicity of definitions, Sandars [16] developed a guide that classifies them into 3 main approaches. According to Sandars’s work [16], reflection is a form of learning that is based on three common aims: (1) reflection for learning, (2) reflection to develop a therapeutic relationship, and (3) reflection to develop professional practice [14,15]. Indeed, reflexivity includes questioning the premises of an action, such as the values, norms, and beliefs that a professional may hold, as well as how such actors justify their actions [17]. In sum, reflexivity is a good strategy for motivating both health care professionals and patients to engage in patient care in a collaborative way.

### Reflexivity and SDM

Reflexivity is appropriate in the context of SDM because the latter emphasizes a partnership between patients and clinicians in making decisions and establishing care plans. SDM aims to reposition the knowledge of patients and clinicians on an equal footing, adjusting the asymmetrical power relationship between patients and health care professionals [18,19]. A prerequisite for implementing SDM in care settings is that health care professionals not only have the knowledge and skills but also the willingness to engage patients in the decision-making process [20].

Training based on reflexivity may lead health care professionals to question the power issues inherent in a more traditional conception of the health system and of their role and may motivate them to adopt SDM [11]. Thus, we hypothesized that SDM training programs that integrate reflexivity strategies would be more effective in increasing the adoption of SDM than those that do not.

### Interprofessional Training

Research also shows that SDM training programs developed with an interprofessional orientation are to be encouraged [21]. Interprofessionality is defined by D’amour et al [22] as the development of a cohesive practice between professionals from different disciplines for the care of a single patient. Specifically, interprofessional collaboration involves collegial, authentic, constructive, open and honest communication as well as mutual trust and respect between professionals who are committed to achieving a common set of goals. However, although an interprofessional orientation is highly encouraged in SDM because of the need for better teamwork and professionals’ openness to other forms of practices, few training sessions with an interprofessional orientation are available [10]. In addition, there is little evidence that interprofessionally oriented training is more effective than other training approaches. Given the similarity between the methods used in training programs with an interprofessional orientation and those used in training programs with certain reflexivity strategies (eg, peer-to-peer group learning), it is important to examine how effective both these training types are.

### Outcome Reporting

In the Cochrane review, one of the criteria for selecting programs was that the type of measurement followed must be a patient-reported outcome measurement (PROM) or an observer-reported outcome measurement (OBOM). A PROM is an instrument used to collect information directly from patients. PROMs do not require amendments or interpretation by a clinician or another observer [23]. An OBOM is any instrument used by a third-party observer to report observable concepts such as signs or behaviors to assess, for example, the decision-making process during an encounter between a patient and family and their health care professional when facing a health treatment or screening decisions [23]. It seemed useful to see what types of measurements were most common, especially among the effective training programs that used reflexivity strategies. This could offer another potential reason for a training program’s effectiveness. As we were interested in interventions targeting health care professionals, it was important to discern whether the type of measurement used to evaluate the intervention was equipped to examine the different levels of effectiveness as defined by Kirkpatrick, the creator of one of the most common evaluation models for assessing training programs targeting health care professionals [24]. The Kirkpatrick model evaluates training programs based on 4 categories: the satisfaction of the participants with the training, improvement of their knowledge, improvement of their care practices, and improvement of patient health. The first category is very important in the evaluation process because, according to Kirkpatrick, learner appreciation is a key factor in motivating participants to learn from training and apply what they learned.

Therefore, based on our analysis of the Cochrane review of interventions for increasing the use of SDM by health care professionals, we first sought to determine whether the SDM training programs for health care professionals that used reflexivity strategies were more frequently classified as effective than the training programs that did not. Second, if such programs were more effective, we aimed to explore the strategy (peer-to-peer group learning or self-appraisal individual learning) that seemed to be more effective. Third, we aimed to examine whether training programs with an interprofessional orientation tended to be evaluated as effective. Finally, we examined whether the type of measurement used (OBOM or PROM) made it possible to classify the training programs in terms of effectiveness.

## Methods

### Study Design

We performed a secondary analysis of a published 2018 Cochrane review that aimed to evaluate the effectiveness of interventions for increasing the use of SDM by health care professionals [7]. As there are no reporting guidelines for the secondary analyses of systematic reviews, we used the PRISMA (Preferred Reporting Items for Systematic Reviews and Meta-Analyses) 2020 for all applicable items [25]. This study took place from February 2021 to April 2022.

### Data Sources and Search Strategy

The search strategy for the Cochrane review serving as the basis of the current secondary analysis was launched on June 15, 2017. Details of the Cochrane search strategy and data sources can be found in the published review [7]. The Cochrane review [7] included interventions classified according to the three target categories of the Effective Practice and Organization of Care (EPOC) taxonomy of interventions [26]: (1) interventions targeting patients (eg, patient-mediated interventions), (2) interventions targeting health care professionals (eg, distribution of printed educational material, educational meetings, audit and feedback, reminders, and educational outreach visits), and (3) interventions targeting both patients and health care professionals (eg, a patient-mediated intervention combined with an intervention targeting health care professionals).

### Eligibility Criteria

In the published Cochrane review, the participants in the training programs could be any type of health care professional (eg, physicians, nurses, pharmacists, or social workers), including professionals in training (eg, resident physicians). Studies that recruited eligible health care professionals along with other types of participants (eg, patients and managers) were also included, as were training programs evaluating a multicomponent intervention (eg, SDM training for health care professionals with the use of patient decision aids). All types of training formats were incorporated into the review (eg, in class, group workshop, web-based training, and synchronous or asynchronous training). A training program was defined as a capacity-building activity conducted live for a group or a single individual, such as a web-based course or a traditional course (ie, a course integrated into an academic program), that used a recognized instructional method such as lectures, workshops, case studies, demonstrations, role plays, and small group discussions [10]. There were no restrictions on the comparison groups, which were all included.

The same primary outcomes of interest reported in the Cochrane review, namely SDM outcomes, were maintained in this secondary analysis, as were the outcome measurements used [7]. The Cochrane review grouped secondary outcomes into 2 categories: patient outcomes (eg, affective-cognitive outcomes, behavioral outcomes, and health outcomes) and process outcomes (eg, consultation length, costs, and equity). The 4 eligible study designs were randomized controlled trials, nonrandomized controlled trials, controlled before-and-after studies, and interrupted time series.

In this secondary analysis, we selected all interventions targeting health care professionals from the Cochrane review (43/87, 49%), excluding interventions that only targeted patients (44/87, 51%). Of the 43 that targeted health care professionals, we excluded interventions that were not a training program (12/87, 14%), for example, demonstrations on how to use a decision aid. Therefore, the total number of articles included in this study was 31.

### Study Selection Process

One of the reviewers (NTD) examined all the content available concerning the interventions used in each of the 87 articles included in the Cochrane review and selected interventions that met the inclusion criteria [7]. Another reviewer (JL) reviewed both the selected and excluded programs to validate the rigor of the selection process. Finally, all the articles involving an eligible training program targeting health care professionals were selected (N=31) (refer to the eligibility criteria section).

### Data Extraction Process

Data extraction was performed by 2 pairs of independent reviewers (NTD and AM or NTD and VB). Differences between the 2 reviewers were resolved by consensus based on discussion and by referring to the definitions provided in the extraction grid (Multimedia Appendix 1). For the remaining conflicts, a third reviewer (SG-B or MCT) intervened to facilitate a consensus. The articles believed to include reflexivity were submitted to MCT for validation. Data extracted included (1) article and study characteristics—year, country and language of publication, and measurement of study design and type; (2) information on the training program—country, training language, context of care, type of health care professional trained, and training format (eg, unidirectional or interactive); and (3) the use or otherwise of reflexivity strategies for interactive training programs and, among those including reflexivity strategies, the type of strategy selected (peer-to-peer reflective group learning or self-appraisal learning).

### Classification of Articles

#### Classification of Training Formats

First, 2 reviewers (NTD and AM or NTD and VB) classified all the included articles (n=31) according to training format (unidirectional or interactive). The interactive training programs were then classified into 2 groups: programs using reflexivity strategies and programs that do not (refer to the details given in the Reflexivity Strategies Assessment section). On the basis of how reflexivity is defined in the literature, in this analysis, only interactive training programs were considered to have the potential to involve reflexivity. The training programs classified here as unidirectional were those in which the trainer delivered the whole message without asking for learner input other than questions, whereas interactive training programs are delivered in a 2-way manner, requiring the active contribution of learners (ie, the trainer delivers the information to the learners and encourages them to contribute to an exchange process). Interactive training by its very nature has the potential for reflexivity through, for example, role play or case discussions. For the purposes of this study, training programs using reflexivity strategies involve at least a minimal contribution from learners in the reflection process. Second, training programs classified as including reflexivity strategies were also categorized into 2 further groups (peer-to-peer group learning or self-appraisal individual learning) by 2 reviewers (NTD and AM or NTD and VB, validated by MCT).

#### Reflexivity Strategies Assessment

To the best of our knowledge, there is no validated set of criteria that defines the minimal components required to qualify a training program as reflexivity based. Thus, we developed a grid informed by a preliminary rapid literature review that synthesized the most common approaches and concepts related to reflexivity strategies used in health care professional training [27]. The grid contains minimal criteria that a training program must meet to be considered as including reflexivity strategies. The 2 following questions (A and B) from our grid were used to assess whether an interactive training program incorporated reflexivity strategies.

#### (A) Does the Training Program Include Any Reflexivity Approaches?

A reflexivity training approach could be, but is not limited to, the following: group-based reflections with peers (with or without a trainer), self-competence improvement with case-based reflections, electronic platforms with reflective portfolios, reflective journals, Balint groups, on-site reflective writing exercises, and the like. When these approaches were not clearly specified, we looked for the common reflexivity concepts.

#### (B) Does the Training Program Include Any Common Reflexivity Concepts?

The following were considered reflexivity concepts: critical thinking, metacognition, self-reflection, reflective dialogue, reflection-in-action, reflection-in-practice, reflection-on-action reflection-on-practice, reflective practice, reflective learning, reflective approaches, reflective dialogues, critical self-reflection, reflective thinking, reflection on error, and the like.

Once we identified articles that included reflexivity strategies based on questions A (presence of reflexivity approaches) and B (presence of reflexivity concepts), we further subcategorized them according to 2 types of strategies: peer-to-peer group learning or self-appraisal individual learning.

#### Peer-to-Peer Group Learning

The main objective of peer-to-peer group learning (small or large group) is to stimulate interaction between participants. In peer-to-peer groups, the participants learn from each other’s reflections while being supported by experienced trainers or facilitators. This strategy can be organized in different ways, for example, a few days of practice followed by a day of reflection among peers or presentation of a topic followed by a group reflection among professionals during which they discuss their practice experience. Various approaches such as reflective writing exercises or groups with colleagues can be incorporated. Reflections may be based on real cases (ie, cases seen in practice) or fictitious ones. Everyone is called upon to give their point of view, and lessons are learned as the reflection progresses [28]. During and at the end of the exercise, the trainer or facilitator reframes the interactions, guides the discussion, and corrects errors or discrepancies resulting from the reflection [28].

#### Self-appraisal Individual Learning

A self-appraisal individual learning strategy is any individual learning process in which the learner is subjected to reflection exercises or cases to be solved. The learner can also be questioned on their practice. The exercise might involve traps to allow learners to detect their own errors. This exercise is performed individually, for example, in a reflective journal where professionals might write down reflections on their practice such as all the events (positive or negative) experienced, what these events meant for them, and what they learned from these experiences [29]. They may then reflect on how such experiences could help them in similar future circumstances. This exercise can also be performed during a group training session but where participants reflect individually on a case (fictitious or real) [30]. A self-appraisal individual learning strategy can also be applied during web-based courses. In a self-appraisal individual learning process, exercises such as self-reflection; reflective learning; reflection on one’s own values, beliefs, and thoughts; and metacognition are often used.

### Assessing Training Effectiveness

We determined whether the included training programs were classified as effective in the Cochrane review [7]. The same outcomes of interest reported in the Cochrane review, that is, SDM outcomes, were maintained in this secondary analysis.

### Analysis

First, unidirectional and interactive training programs were compared to see what percentage of each was classified as effective by the Cochrane review. Second, among the interactive programs, those that used reflexivity strategies were compared with those that did not to see which were more frequently effective. Third, reflexivity strategies (peer-to-peer group learning and self-appraisal individual learning) were compared. After these 3 steps, we carried out an additional analysis to see whether there were elements that could explain why a training program was effective or otherwise, apart from those cited earlier. For example, we compared the proportion of training programs based on interprofessional orientation that were effective with the proportion of programs using reflexivity that were effective. In addition, to see how SDM training programs can be better evaluated, we classified training programs according to the type of measurement they incorporated and whether they were classified as effective.

## Results

### General Characteristics of Results

#### Main Characteristics of the Included Studies

**Figure 1:**
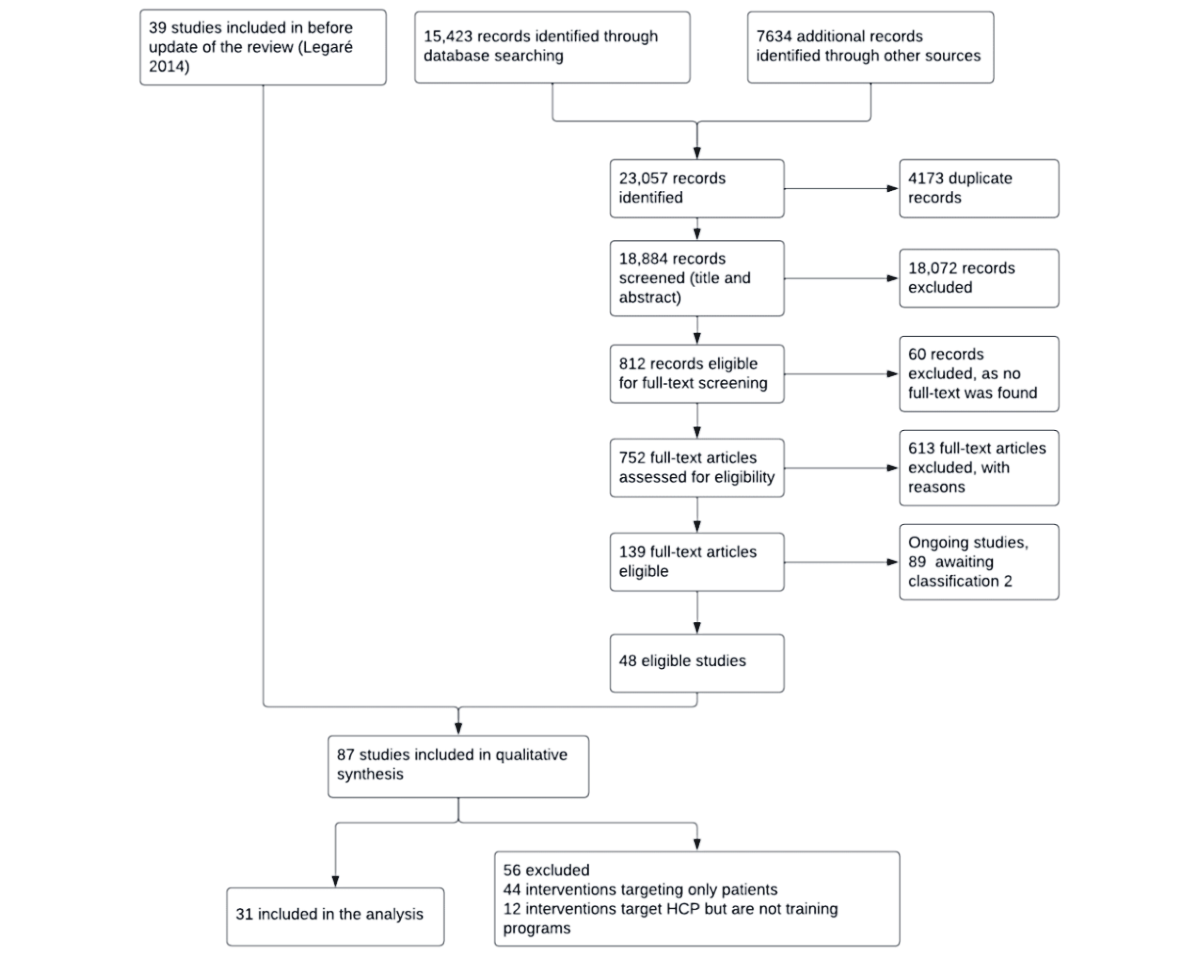
Selection process of the included studies. HCP: health care professional.

All the 87 studies included in the Cochrane review were evaluated [7]. Of these, 43 (49%) interventions targeted health care professionals, and 44 (51%) targeted only patients. Among the 43 interventions targeting health care professionals, 31 (72%) were found to be training programs (Figure 1) and were analyzed in this study.

Of the 31 articles included, 10 (32%) were published between 2002 and 2010 (the first publication of the Cochrane review) [31-40], 14 (45%) were published between 2011 and 2014 (the first update of the Cochrane review) [41-54], and the other 7 (23%) were published between 2015 and 2017 (the most recent update) [55-62]. A total of 11 (35%) included articles were published in the United States [38, 43, 46, 50, 51, 53-55, 58, 60, 61, 63], followed by 8 (26%) in Germany [3,32,35,37,39,44,47,57,64-66] (Table 1). All the studies included were published in English, and 29 (94%) of them were RCTs. For the primary outcome assessment, 15 (48%) of the 31 studies were evaluated using PROMs alone, 12 (39%) were evaluated using OBOMs, and 3 (10%) were evaluated using both. Information about the 1 (3%) study [35] that used health care professional–reported outcome measurements (HCPROMs) was directly collected from the article. Cochrane did not include outcomes measured by HCPROMs in its analysis [7]. However, seeing that this paper [35] was included in the analysis of some secondary outcomes in the Cochrane review, we examined the results directly from the article to analyze information related to our variable of interest (SDM).

**Table 1:** Characteristics of included studies (N=31).

Characteristics		Values, n (%)
**Year of publication**		
	2002-2010	10 (32)
	2011-2014	14 (45)
	2015-2017	7 (23)
**Country**		
	United States	11 (35)
	Germany	8 (26)
	United Kingdom	4 (13)
	Canada	3 (10)
	Norway	2 (6)
	Other (Netherlands, Belgium, and Switzerland)	3 (10)
**Language**		
	English	31 (100)
**Study design**		
	Randomized controlled trials	29 (94)
	Nonrandomized controlled trials	1 (3)
	Before-and-after studies	1 (3)
**Outcome measure assessors**		
	PROM^a^	15 (48)
	OBOM^b^	12 (39)
	PROM and OBOM	3 (10)
	PROM and HCPROM^c^	1 (3)

^a^PROM: patient-reported outcome measurement.

^b^OBOM: observer-reported outcome measurement.

^c^HCPROM: health care professional–reported outcome measurement.

#### Main Characteristics of the Training Programs

Of the 31 training programs, 11 (35%) were developed in the United States, and 8 (26%) in Germany (Table 2). A total of 22 (71%) programs were in English, followed by 4 (13%) in German and 2 (6%) in Dutch. Of the remaining programs, 1 (3%) was in French, 1 (3%) was in both English and Spanish, and language was not reported for one of the programs. Of the 31 training programs, 20 (65%) were developed in a primary health care context, and 11 (35%) were developed in specialized care. Regarding the type of health care professionals trained, 18 (58%) targeted physicians, and 26 (84%) were developed for fully trained health care professionals (Table 2). Only 5 (16%) training programs out of the 31 were developed with an interprofessional orientation, that is, with the promotion of interprofessionality as one of its training objectives.

A total of 24 (77%) of the 31 programs had an interactive format [3,32-39,41-45,51-60,64], and the remaining 7 (23%) were unidirectional training programs [40,46-50,61]. Among the 24 interactive training programs, 7 (29%) were developed using reflexivity strategies [32-34,41,42,55,56,67,68], among which 5 (71%) were classified as peer-to-peer group learning [32,33,41,42,56,64,69] and 2 (29%) as self-appraisal individual learning [34,55,62]. Details on how the training programs were classified according to the reflexivity strategy used are reported in Table 3.

**Table 2:** Training program characteristics (N=31).

Characteristics		Values, n (%)
**Country**		
	United States	11 (35)
	Germany	8 (26)
	United Kingdom	4 (13)
	Canada	3 (10)
	Norway	2 (7)
	Australia, Netherlands, Switzerland, and Germany (collaboration)	1 (3)
	Others (Netherlands and Belgium)	2 (7)
**Language**		
	English	22 (71)
	German	4 (13)
	Dutch	2 (7)
	French	1 (3)
	English and Spanish	1 (3)
	Not reported	1 (3)
**Context of care**		
	Primary care	20 (65)
	Specialized care	11 (36)
**Types of professional trained**		
	Physicians^a^	18 (58)
	Nurses and geneticists	8 (26)
	Nurses	2 (7)
	Physicians and nurses	2 (7)
	Physicians and midwives	1 (3)
	Fully trained	26 (84)
	Not fully trained	5 (16)
Interprofessional orientation		5 (16)

^a^One training program was designed for oncologists and gynecologists, and the other included medical residents.

**Table 3:** Quotations illustrating how reflexivity strategies are reported.

Articles	Main quotations	Types of reflexivity strategies
Krones et al [32], 2008	“Practical communication strategies” “*After role-play feedback was given by their peers*”^a^ “Educational outreach...members *were invited to moderate the sessions*” “Using the script-like decision aid was practiced through role playing” “Participants *received feedback from peers in their groups*”	Peer-to-peer reflective group learning
Murray et al [33], 2010	“Self and *peer appraisal during role play*” “Participants will evaluate decision-support skills to *self-appraise* their own and *workshop peers’ quality of decision support during the case studies and role-play activities*” “To train nurses and medical residents in *self-appraisal*”	Peer-to-peer reflective group learning
Sanders et al [56], 2017	“*The training was based on the learning principles described by Kolb*. In the training sessions, *group discussion*, theory, *role-playing, and reflections on personal behaviour were alternated*. This tool was generated in the first session when *the GPs reflected on their training experiences*”	Peer-to-peer reflective group learning
Fossli et al [41], 2011	“The course consisted of a 50/50 mix of theory and 45 min group sessions (3-7 participants and *two teachers per group) including role-plays, with plenary debriefs after each group*” “Our course was based on the same content as the 5-day course Communication Skills Intensive offered by Kaiser Permanente” “At the conclusion of the course, all participants received a one-sheet overview of the Four Habits to carry in their pockets as reminder in everyday work”	Peer-to-peer reflective group learning
Kennedy et al [42], 2013	“Skills to encourage a structured approach to self-care support in consultations. Interactive role play (*small groups*) techniques to help deal with difficult issues during consultations. *Interactive role play (small groups). Brief presentation with discussion. DVD exemplar of use plus manual involving (whole group). Explanatory models to encourage discussion about the causes and consequences of long-term conditions... Presentation with discussion*. DVD exemplar of use plus manual (*involving whole group*)... As a practice–develop skills to solve problems that come up in the work of the practice. Problem-solving techniques involving whole practice systems within practice to improve self-care support for patients.” “Problem-solving techniques involving whole practice ways to engage patients with self-care support.”	Peer-to-peer reflective group learning
Elwyn et al [34], 2004	“Practitioners attended two workshops. During the first workshop, the background literature on SDM was outlined and participants were asked to debate its relevance to clinical practice. The skills of SDM were described and demonstrated using simulated consultations. This provided opportunities for all the participants to comment on the method, using an observational competence checklist. Simulated patients were also encouraged to comment. *Participants were asked to consult with the simulated patients using preprepared scenarios involving the study conditions*. At the second workshop, *participants were asked to consider the competences in more depth*. By the end of the workshop, all participants had conducted and *received feedback from at least one consultation with a simulated patient*”	Self-appraisal individual learning
Epstein et al [55], 2017	“A 2-session in-office physician training (1.75 hours) using a brief video, *feedback from standardized patients portraying roles of patients with advanced cancer, audio recorded study patient visits, and (2) (...), plus up to 3 follow-up phone calls* (Table 1).”	Self-appraisal individual learning

^a^Text in italics illustrates possible reflexivity approaches and concepts (related to questions A and B of our criteria grid).

### Effectiveness of the Training Programs in Connection With Different Variables

#### Training Formats and Effectiveness

Based on the Cochrane review classification of the effectiveness of the included interventions, 10 (42%) of the 24 interactive training programs were deemed effective, as opposed to 1 (14%) of the 7 unidirectional programs (Table 4).

**Table 4:** Effectiveness of the training programs according to the measures reported.

Training format and articles			SMD^a^, EMD^b^, or RD^c^ (95% CI)		Narrative results		Effective^d^
**Interactive format (including reflexivity strategies)**							
	Krones et al [32], 2008; Hirsch et al [64], 2010	0.40 (0.28 to 0.52)		N/A^e^		Yes	
	Murray et al [33], 2010	3.75 (2.46 to 5.03)		N/A		Yes	
	Sanders et al [56], 2017	0.85 (0.54 to 1.16); 0.93 (0.62 to 1.25)		N/A		Yes	
	Elwyn et al [34], 2004; Edwards et al [67], 2004; Longo et al [68], 2006	−0.30 (−1.19 to 0.59); 0.05 (−0.17 to 0.27)		N/A		No	
	Fossli et al [41], 2011	0.38 (−0.17 to 0.94)		N/A		No	
	Kennedy et al [42], 2013; Kennedy et al [69], 2010	−0.05 (−0.12 to 0.01)		N/A		No	
	Epstein et al [55], 2017; Butow et al [62], 2015	0.00 (−0.24 to 0.24)		N/A		No	
**Interactive format (not including reflexivity strategies)**							
	Bieber et al [35], 2006; Bieber et al [65], 2007	N/A		“An ANOVA for repeated measurements comparing the SDM group with the information group revealed that patients’ appraisal of the interaction quality was higher in the SDM group”		Yes	
	Stacey et al [36], 2008	2.07 (1.26 to 2.87)		N/A		Yes	
	Loh et al [37], 2007	N/A		“In the intervention group, significantly higher patient participation from pre- to postintervention was found.”		Yes	
	Haskard et al [38], 2008	N/A		“Training significantly improved physicians’ health behaviour counseling of their patients.”		Yes	
	Deinzer et al [39], 2009	N/A		“The degree of SDM was significantly higher in the SDM group at baseline and after one-year visits. The results of the SDM sum score on actually practiced SDM exhibited in both groups significantly increased, but the control group did not reach the score of the study group after one year.”		Yes	
	Feng et al [43], 2013	N/A		“Significant difference in favour of the intervention group, high risk of bias.”		Yes	
	Tinsel et al [44], 2013	0.32 (0.17 to 0.46)		N/A		Yes	
	Hamann et al [3], 2007	0.16 (−0.28 to 0.61)		N/A		No	
	Bernhard et al [45], 2011; Butow et al [62], 2015	N/A		“There was no effect for this variable for SGA^f^ doctors (estimated population mean difference 0.52, SE 1.39, ES^g^=0.04; *P*=.71)” “After the training workshop, doctors in the experimental group within the ANZ^h^ cohort displayed more behaviours designed to establish the SDM framework than doctors in the control group (estimated population mean difference=3.42, SE 1.50, ES=0.30, *P*=.03). However, the ES was small” “There was considerable variation in patient outcomes between the SGA and ANZ cohorts and no substantial training effect”		No	
	Cooper et al [54], 2011	0.11 (−0.30 to 0.51); 0.03 (−0.15 to 0.20); 0.16 (−0.23 to 0.56)		N/A		No	
	Légaré et al [52], 2012; Allaire et al [70], 2012; Légaré et al [71], 2013	0.01 (−0.03 to 0.06)		N/A		No	
	Cooper et al [53], 2013	0.70 (0.30 to 1.90)^5^		N/A		No	
	Wilkes et al [51], 2013	−0.13 (−0.32 to 0.05)		N/A		No	
	Härter et al [57]^i^, 2015; Bieber et al [66], 2018	0.54 (0.35 to 0.74); −0.07 (−0.26 to 0.12); 0.11 (−0.10 to 0.31)		N/A		No	
	Tai-Seale et al [58]^j^, 2016; Dillon et al [63], 2017	0.35 (−0.53 to 1.24); 0.51 (0.19 to 0.84); −0.29 (−1.17 to 0.60); 0.00 (−0.32 to 0.32)		N/A		No	
	Ampe et al [59], 2017	−0.10 (−0.96 to 0.76)		N/A		No	
	Cox et al [60], 2017	0.11 (−0.21 to 0.42)		N/A		No	
**Unidirectional**							
	Hess et al [46], 2012	2.82 (2.43 to 3.21)		N/A		Yes	
	O’Cathain et al [40], 2002	−0.02 (−0.05 to 0.01)		N/A		No	
	Koerner et al [47], 2014	−0.08 (−0.26 to 0.11)		N/A		No	
	Mathers et al [48], 2012	−0.09 (−0.23 to 0.05)		N/A		No	
	Rise et al [49], 2012; Rise et al [72], 2016	0.13 (−0.32 to 0.58)		N/A		No	
	Sheridan et al [50], 2014	−0.17 (−0.35 to 0.00)		N/A		No	
	Coylewright et al [61], 2016	0.51 (−0.05 to 1.07)		N/A		No	

^a^SMD: standardized mean difference.

^b^EMD: effect size mean difference.

^c^RD: risk difference.

^d^Scored as “Yes” if the 95% CI reported in the Cochrane review did not include 0 for the SMD, RD, and MD values or when the 95% CI did not include 1 for the OR values. In some studies, with ≥2 scales, we referred to the conclusion of the authors.

^e^N/A: not applicable.

^f^SGA: Switzerland, Germany, and Australia.

^g^ES: effect size.

^h^ANZ: Australia and New Zealand.

^i^This study found no effect of shared decision-making training on the primary outcomes, which were similar between both the groups. However, training did contribute to improved observer-rated shared decision-making skills in physicians and reduced anxiety and depression in patients, particularly in women with breast cancer.

^j^The primary outcome measure was CollaboRATE, a patient-reported experience with care. While the odds ratios (ORs) from the ASK (Ask Share Know) clinic (OR 1.417) and the OpenComm plus ASK clinic (OR 1.134) were greater than 1, their 75% CIs included 1, which suggests no difference from the usual care clinic. Our findings suggest that something could be done to improve the patient experience. We view the results as promising evidence of the intervention’s efficacy and as meaningful signals of its likely effects on patient experience.

#### Reflexivity Strategies and Effectiveness

Regarding the effectiveness of the programs, 3 (43%) of the 7 programs including reflexivity strategies were deemed effective [32,33,56]. The number of effective programs among programs with reflexivity strategies was similar to that among interactive programs without reflexivity strategies. Concerning the latter, 7 (41%) out of 17 were deemed effective (Table 4) [35-39,43,44].

Table 4 shows that 3 (60%) of the 5 training programs using a peer-to-peer group learning strategy were effective [32,33,56], whereas none of those using a self-appraisal individual learning strategy were effective (Table 4).

#### Interprofessional Approach and Effectiveness

Of the 5 training programs developed with an interprofessional orientation, 3 (60%) were classified by the Cochrane review as effective [32,33,46], and 2 (67%) of these included reflexivity strategies [32,33] (Table 5). In other words, the 2 programs with an interprofessional orientation that included reflexivity strategies were both deemed effective.

**Table 5:** Interprofessional orientation and type of measurement.

Studies	Effective	IP^a^ approach	Outcome assessors	Training format
Krones et al [32], 2008; Hirsch et al [64], 2010	Yes	Yes	PROM^b^	Interactive format (including reflexivity strategies)
Murray et al [33], 2010	Yes	Yes	OBOM^c^	Interactive format (including reflexivity strategies)
Sanders et al [56], 2017	Yes	No	OBOM OBOM	Interactive format (including reflexivity strategies)
Elwyn et al [34], 2004; Edwards et al [67], 2004; Longo et al [68], 2006	No	No	OBOM PROM	Interactive format (including reflexivity strategies)
Fossli et al [41], 2011	No	No	OBOM	Interactive format (including reflexivity strategies)
Kennedy et al [42], 2013; Kennedy et al [69], 2010	No	No	PROM	Interactive format (including reflexivity strategies)
Epstein et al [55], 2017; Butow et al [62], 2015	No	No	PROM	Interactive format (including reflexivity strategies)
Bieber et al [35], 2006; Bieber et al [65], 2007	Yes	No	PROM HCPROM	Interactive format (not including reflexivity strategies)
Stacey et al [36], 2008	Yes	No	OBOM	Interactive format (not including reflexivity strategies)
Loh et al [37], 2007	Yes	No	PROM	Interactive format (not including reflexivity strategies)
Haskard et al [38], 2008	Yes	No	OBOM	Interactive format (not including reflexivity strategies)
Deinzer et al [39], 2009	Yes	No	OBOM	Interactive format (not including reflexivity strategies)
Feng et al [43], 2013	Yes	No	OBOM	Interactive format (not including reflexivity strategies)
Tinsel et al [44], 2013	Yes	No	PROM	Interactive format (not including reflexivity strategies)
Hamann et al [3], 2007	No	No	PROM	Interactive format (not including reflexivity strategies)
Bernhard et al [45], 2011; Butow et al [62], 2015	No	No	OBOM	Interactive format (not including reflexivity strategies)
Cooper et al [54], 2011	No	No	PROM PROM PROM	Interactive format (not including reflexivity strategies)
Légaré et al [52], 2012; Allaire et al [70], 2012; Légaré et [71], 2013	No	No	PROM	Interactive format (not including reflexivity strategies)
Cooper et al [53], 2013	No	No	PROM	Interactive format (not including reflexivity strategies)
Wilkes et al [51], 2013	No	No	PROM	Interactive format (not including reflexivity strategies)
Härter et al [57], 2015; Bieber et al [66], 2018	No	No	PROM OBOM OBOM	Interactive format (not including reflexivity strategies)
Tai-Seale et al [58], 2016; Dillon et al [63], 2017	No	No	OBOM PROM OBOM PROM	Interactive format (not including reflexivity strategies)
Ampe et al [59], 2017	No	No	OBOM	Interactive format (not including reflexivity strategies)
Cox et al [60], 2017	No	Yes	OBOM	Interactive format (not including reflexivity strategies)
Hess et al [46], 2012	Yes	Yes	OBOM	Unidirectional
O’Cathain et al [40], 2002	No	No	PROM	Unidirectional
Koerner et al [47], 2014	No	Yes	PROM	Unidirectional
Mathers et al [48], 2012	No	No	PROM	Unidirectional
Rise et al [49], 2012; Rise et al [72], 2016	No	No	PROM	Unidirectional
Sheridan et al [50], 2014	No	No	PROM	Unidirectional
Coylewright et al [61], 2016	No	No	OBOM	Unidirectional

^a^IP: interprofessional.

^b^PROM: patient-reported outcome measure.

^c^OBOM: observer-reported outcome measure.

#### Outcome Assessors and Effectiveness

Among the selected articles, based on the Cochrane review, 8 (67%) out of the 12 programs using OBOMs were classified as effective [33,36,38,39,43,46,56], while 3 (21%) of the 14 programs using PROMs were classified as effective [32,44]. Meanwhile, 2 (67%) of the 3 training programs that included reflexivity strategies and were classified as effective were assessed using OBOMs. The only effective program in the unidirectional category was assessed using OBOMs (Table 5).

The main findings related to the different elements analyzed in the study are summarized in Table 6.

**Table 6:** Summary of main findings (N=31).

Level of analysis and training categories							Training programs							Effective program							IP^a^ approach							Effective program with IP approach							Type of assessment
						Values, N				Values, n (%)			Values, N				Values, n (%)			Values, N		Values, n (%)					Values, N				Values, n (%)				
	**Level 1**																																		
			**Format**																																
				Interactive	31			24 (7)			24				10 (42)			5					3 (60)		3				2 (67)			OBOM^b^: 10/24 (42); PROM^c^: 10/24 (42); PROM/OBOM: 3/24 (12); PROM/HCPROM: 1/24 (4)			
				Unidirectional	31			7 (23)			7				1 (14)			5					2 (40)		2				1 (50)			OBOM: 2/7 (29); PROM: 5/7 (71)			
**Level 2**																																			
		**Interactive**																																	
				Reflexivity strategies—yes	24				7 (30)			7				3 (43)			3					2 (67)		3				2 (67)			OBOM: 3/7 (43); PROM: 3/7 (43); PROM/OBOM: 1/7 (14)		
				Reflexivity strategies—no	24				17 (70)			24				7 (30)			3					1 (33)		1				0 (0)			OBOM: 7/17 (41); PROM: 7/17 (41); PROM/OBOM: 2/17 (12); PROM/HCPROM: 1/17 (6)		
**Level 3**																																			
		**Reflexivity strategies—yes**																																	
				Peer-to-peer group learning	7				5 (71)			5				3 (60)			2					2 (100)		2				2 (100)			OBOM: 3/5 (60); PROM: 2/5 (40)		
				Self-appraisal learning	7				2 (29)			5				0 (0)			2					0 (0)		N/A^d^				N/A			PROM: 1/2 (50); PROM/OBOM: 1/2 (50)		

^a^IP: interprofessional

^b^OBOM: observer-reported outcome measure.

^c^PROM: patient-reported outcome measure.

^d^N/A: not applicable.

### Risk of Bias Assessment

The risk of bias assessment was reported in the initial Cochrane review, which used criteria for EPOC reviews [73] and the *Cochrane Handbook for Systematic Reviews of Interventions* [74] for interrupted time series designs.

### Certainty of Evidence

For our variable of interest (the primary outcome), the certainty of evidence was assessed in the Cochrane review according to the GRADE (Grading of Recommendations Assessment, Development and Evaluation) guidelines and methods described in Chapter 12 of the *Cochrane Handbook for Systematic Reviews of Interventions* [75]. The method proposed in EPOC worksheets was used to determine which secondary outcomes should be assessed [76].

## Discussion

### Principal Findings

This secondary analysis of a 2018 Cochrane review on interventions for increasing the use of SDM by health care professionals aimed to identify training programs that included reflexivity and to ascertain how effective they were. Our study is the first to evaluate the effectiveness of SDM training programs that include reflexivity strategies. Among the 31 SDM training programs for health professionals, 23% (n=7) included reflexivity and 77% (n=24) did not. More of those that included reflexivity were deemed effective as a percentage of the whole (3/7, 43%) than those that did not (8/24, 33%). Among the interactive training programs, there was little difference in effectiveness between those that used reflexivity strategies (3/7, 43%) and those that did not (7/17, 41%). However, when comparing interactive training programs with unidirectional ones, there were a great many more programs deemed effective among the former (10/24, 42%) than among the latter (1/7, 14%).. Among the training programs that included reflexivity (n=7), most programs using a peer-to-peer group learning strategy were found to be effective (3/5, 60%), whereas those using a self-appraisal individual learning strategy were not (0/2, 0%). The training programs with an interprofessional orientation were more frequently classified as effective (3/5, 60%) than those without (2/5, 40%). Finally, the percentage of effective training programs in studies using OBOMs to assess training was higher than the percentage in studies using PROMs. These findings led to the observations that follow.

### A Larger Percentage of Reflexivity-Based Training Programs Were Deemed Effective

Our results confirm the findings of Leyland et al [77] and Chaffey et al [12] that in general, programs that include reflexivity have more positive effects than those that do not [12,77]. Training using reflexivity strategies has been shown to increase medical students’ ability to integrate alternative sources of knowledge and critically reflect on their own practices [12]. According to Chaffey et al [12], it is difficult to assess reflexivity alone, but when applied in an intervention, it yields more positive results. Although only a few SDM training programs include reflexivity strategies to date, these results suggest that reflexivity could be a core component of effective training and, as such, may be understood as an effective implementation strategy for change. However, further studies with a larger sample are needed to confirm this hypothesis. For Kolb [78], learning is a complex process driven partly by individuals’ ability to find a sense for themselves in operationalizing change in their day-to-day practices. As our results suggest, training that uses reflexivity strategies could provide health care professionals with that personal sense of the purpose of SDM and the motivation needed to implement SDM in their practices. However, future research could more precisely evaluate the impact of reflexivity strategies on SDM uptake by health care professionals if more programs were explicit in their use or promotion of reflexivity. The training programs included in this review were not designed to use or promote reflexivity. An analysis that compared SDM training programs that were explicitly based on reflexivity with those that were not would be more useful and appropriate. Nevertheless, our results suggest that SDM training for health care professionals based on reflexivity strategies is effective in motivating trainees to adopt SDM in a manner that facilitates positive patient experiences in health care systems.

### A Peer-to-Peer Group Learning Strategy Would Appear to Be More Effective Than a Self-appraisal Learning Strategy

Our findings also showed that more training programs using peer-to-peer group learning were classified as effective than those using self-appraisal individual learning. These findings suggest that a self-appraisal strategy can be more effective than training focused on an individual if it is part of a group learning process. Interaction among learners seems to be a powerful strategy for encouraging reflection, even self-reflection. Research could further compare the types of reflexivity learning strategies that are most effective in SDM training for health care professionals.

### Training Using Reflexivity Strategies and an Interprofessional Orientation Can Lead to Better Results

Our findings indicated that training programs with an interprofessional orientation have proven to be more effective than those without this orientation. All programs that used interprofessional orientation and reflexivity strategies were also deemed effective. In addition, these programs used a peer-to-peer group learning strategy in which participants were encouraged to learn from each other’s reflections by sharing their points of view and experiences [28]. One of the main goals of interprofessional SDM is to encourage the recognition of other professionals’ values and competencies [21,79], in other words, to recognize other members of the care team as peers [22]. Leyland et al [77] also considered that reflexivity is needed for health care professionals to recognize each other’s professional skills. This suggests that the peer-to-peer group learning strategy is especially appropriate for implementing an interprofessional approach. In addition, Tremblay et al [27] defined the goal of “formative reflexivity” (ie, reflexive practices for learning) as developing new visions of professional experience and working in collaboration. Thus, blending these 2 goals (interprofessional orientation and reflexivity) is a promising avenue for fostering the uptake of interprofessional SDM in health practice. Furthermore, encouraging reflection and questioning of one’s practice can help to correct the paternalistic way in which SDM still seems to be undertaken [19,77].

### Measuring SDM Training for Health Care Professionals Only With OBOM or PROM, a Limiting Approach

The Cochrane review used only OBOMs and PROMs (or both) to evaluate intervention effectiveness, eschewing programs that used HCPROMs. In the review, most programs classified as effective were assessed using OBOMs (8/11, 73%) rather than PROMs (3/11, 27%). OBOMs are considered a more rigorous assessment option than self-reported measures because they are more independent [23]. Based on this justification, one can say that the apparent objectivity of OBOM assessment yields better results than the potential subjectivity arising when patients report an outcome. Furthermore, our findings showed that 2 (67%) of the 3 training programs using reflexivity and classified as effective were assessed with OBOMs, as opposed to only 1 (33%) with PROMs. However, neither PROMs nor OBOMs respect the first Kirkpatrick model criterion, which is the satisfaction of the participants with the training. According to Kirkpatrick, when participants like a training program, they may be willing to adopt what they have learned from it. Based on the importance of this first level of evaluation, we suggest that the perspective of the training beneficiaries (health care professionals) should be considered in evaluations, as is the case with HCPROMs. This does not exclude the use of PROM or OBOM measures to assess Kirkpatrick’s other 3 levels of improvement: knowledge improvement, practice improvement, and health-related outcome improvement (the levels considered in the Cochrane review). Furthermore, a reflexivity-based approach focuses not only on outcomes but also on the process or experience of health care, which may be as important to patients as outcomes [80]. If trainees reflect on their own practice and question their own values, they will better consider patients’ values and experiences in the health care-seeking process. Therefore, another relevant measure of effectiveness would be patient-reported experience measures (PREMs), which help improve patient care [81]. In a 2019 systematic review, Müller et al [9] analyzed the methods used by 41 studies that assessed SDM training programs for health care professionals and concluded that the diversity of assessment methods limits the ability to compare training program effectiveness and is a barrier to conclusive evidence. Therefore, it now seems important to develop a harmonized SDM training assessment measure that includes all the 4 perspectives to enable better comparison.

### Multiplicity of Elements Did Not Facilitate the Analysis

In this review, some interventions involved multiple components, for example, they included a workshop, a web-based tutorial, and a decision aid tool or a workshop with audit and feedback. In these cases, it was difficult to evaluate the effects of the components separately. In addition, some of the training programs included patients. If the training includes patients, it can be difficult to know whether this inclusion has an additional impact on effectiveness. Another example is the diversity of the comparators. The articles included in the review used different types of comparators (usual care or another differing intervention). Finally, even if the primary outcome (SDM) was our focal interest, it may be defined differently from one study to another. For example, while some focus their analysis on the uptake of SDM as a whole, others may analyze only one component (eg, decision conflict or decision regret) or separate them. In some articles, analysis used ≥2 scales, which made our judgment difficult, especially if effectiveness-related results were different. To classify these types of articles in terms of effectiveness, we referred to the conclusions of the authors. Based on all these observations, we suggest using a core set of assessment methods and validated outcomes for all learning levels in SDM training programs to identify the most effective strategies and better compare them. Future research could explore methods to specifically assess reflexivity strategies included in SDM training programs targeting health care professionals for their inclusion in this core model. Our reflexivity grid, when validated and published, along with the results from this analysis, can be considered as the first step in assessing SDM training that uses reflexivity strategies, but further work is needed to guide training approaches in this field.

### Limitations

This study has a few limitations. First, the systematic review included evidence only up to June 2017, and another update has not been performed since then. The Cochrane review has been updated twice, with each update including all the studies in the earlier versions; yet, its conclusions regarding effectiveness have varied little since the first review in 2010. At its first publication, it included only 5 RCTs, but in 2018, with 84 RCTs, it still concluded that “a great variety of activities exist to increase shared decision-making by health care professionals, but we cannot be confident about which of these activities work best because the certainty (or the confidence) of the evidence has been assessed as very low.”

Second, seeing that the training programs included in the systematic review were not explicit about promoting reflexivity, the selection of those that included what could be accurately described as reflexivity strategies was not an easy task. Nevertheless, using our grid based on a preliminary rapid review, we identified 7 programs. This was a small number for further analysis, although not surprising, as reflexivity is a new concept in the context of health professional education about SDM. It is possible that we missed a few programs that included reflexivity, as we relied solely upon published data and did not contact the authors for additional information. To minimize this risk, we analyzed all additional materials cited in the articles that were linked with the training programs and considered the most recent publications to discuss our results. Finally, the grid used in the analysis was developed for the purpose of this study, and although it is an important contribution to the field of implementation, its validation will require further use and assessment.

### Conclusions

Our results suggest that SDM training programs for health care professionals using reflexivity strategies could increase SDM implementation. Our study is the first to evaluate the effectiveness of SDM training that includes reflexivity and raises several questions: (1) Are peer-to-peer learning strategies more effective than self-appraisal strategies? (2) How can reflexivity and interprofessional orientation strategies best complement each other? (3) Are OBOMs and PROMs the only appropriate means of evaluating SDM training programs? The grid developed for identifying reflexivity strategies in training programs, including reflexivity-related approaches and concepts, will be a useful guide for developing reflexivity training and is to be validated in future studies.

## Appendix

Multimedia Appendix 1 Extraction grid for training program based on reflexivity strategies.

## Abbreviations

EPOC: Effective Practice and Organization of Care
HCPROM: health care professional–reported outcome measurement
OBOM: observer-reported outcome measurement
PREM: patient-reported experience measure
PRISMA: Preferred Reporting Items for Systematic Reviews and Meta-Analyses
PROM: patient-reported outcome measurement
RCT: randomized controlled trial
SDM: shared decision-making
